# Effect of Loading Frequency Ratio on Multiaxial Asynchronous Fatigue Failure of 30CrMnSiA Steel

**DOI:** 10.3390/ma14143968

**Published:** 2021-07-15

**Authors:** Tianqi Liu, Xinxin Qi, Xinhong Shi, Limin Gao, Tian Zhang, Jianyu Zhang

**Affiliations:** 1 School of Aeronautic Science and Engineering, Beihang University, Beijing 100191, China; liutianqi@comac.cn (T.L.); qixin@buaa.edu.cn (X.Q.); 2 Beijing Key Laboratory of Civil Aircraft Structures and Composite Materials, COMAC Beijing Aircraft Technology Research Institute, Beijing 102211, China; liutianqi@buaa.edu.cn (L.G.); qixinxin@buaa.edu.cn (T.Z.); 3 College of Aerospace Engineering, Chongqing University, Chongqing 400044, China; jyzhang@buaa.edu.cn

**Keywords:** 30CrMnSiA steel, crack growth path, fatigue life prediction, asynchronous loading, frequency ratio

## Abstract

Multiaxial asynchronous fatigue experiments were carried out on 30CrMnSiA steel to investigate the influence of frequency ratio on fatigue crack initiation and propagation. Test results show that the surface cracks initiate on the maximum shear stress amplitude planes with larger normal stress, propagate approximately tens of microns, and then propagate along the maximum normal stress planes. The frequency ratio has an obvious effect on the fatigue life. The variation of normal and shear stress amplitudes on the maximum normal stress plane induces the crack retardation, and results in that the crack growth length is longer for the constant amplitude loading than that for the asynchronous loading under the same fatigue life ratio. A few fatigue life prediction models were employed and compared. Results show that the fatigue life predicted by the model of Bannantine-Socie cycle counting method, section critical plane criterion and Palmgren-Miner’s cumulative damage rule were more applicable.

## 1. Introduction

In engineering practice, the multiaxial fatigue failure behavior of structures and components is affected by the phase angle, mean stress, loading sequence, stress/strain amplitude ratio, and frequency ratios of tensile to shear stress/strain components (asynchronous loadings). Compared with other multiaxial loading paths, there are limited studies on multiaxial asynchronous loadings. A lot of researches on steel materials were focused on the fatigue failure under uniaxial and multiaxial constant amplitude loadings, such as the fatigue failure mechanism of 25CrMo4 (EA4T) steel [1,2,3] and 39NiCrMo3 steel [4], crack initiation mechanism of 25CrMo4 steel [5] and 30CrMnSiA steel [6,7], short crack growth behavior of 25CrMo4 steel [8], 42CrMo4 steel [9,10,11] and 30CrMnSiA steel [6,7], the influence of chemical composition on crack initiation of 25CrMo4 steel [12], and the influence of loading paths on fatigue failure of 30CrMnSiA steel [13,14,15,16], while there are limited studies on the fatigue failure mechanism and influencing factors under multiaxial random fatigue loadings.

Multiaxial random fatigue is one of the most common forms of mechanical failure of metal alloys in engineering practice, and asynchronous loading is one of the simplest. In most studies [17,18,19], the asynchronous loading path is usually considered as one type of multiaxial loading path. The commonly used frequency ratios of tensile to shear stress or strain are 2:1 and 1:2 [20,21,22]. Experiments were conducted on S460N steel by Vormwald et al. [20,21] to verify the application of fatigue criterion based on the cyclic plasticity of a material, and the frequency ratios of tensile to shear strain are 2:1 and 1:2 with a stress amplitude ratio of $\sqrt{3}$. Noban et al. [22] studied the fatigue characterization of 30CrNiMo8HH steel under multiaxial asynchronous loadings with a frequency ratio of 1:2 and the stress amplitude ratio used was $1/\sqrt{3}$. A lot of researches focused on the influence of the frequency ratio on fatigue limit and similar conclusions were drawn that the fatigue limit decreases when the tensile or shear frequency ratio increases, such as the Mielke’s study [23] on 25CrMo4 steel with the frequency ratios of 4:1, 1:1, 1:2 and 1:8, Heidenreich’s study [24] on 34Cr4 steel with the frequency ratios of 4:1, 1:1 and 1:4, McDiarmid’s study [25,26,27] on EN24T steel with the frequency ratios of 1:1, 1:2 and 1:3 and Bernasconi’s study [28] on 39NiCrMo3 steel with the frequency ratios of 1:1, 1:2 and 1:3. Some other studies focused on the crack initiation and propagation behavior under asynchronous loading. Experiments using 42CrMo4 steel were carried out by Reis et al. [29], and the frequency ratios were 2:1 and 1:2 with a constant stress amplitude ratio of $1/\sqrt{3}$. By using the shear-based models, the direction of crack initiation was well predicted. In order to study the effect of asynchronous load on the stress-strain response of materials, four materials including aluminum alloy, austenitic stainless steel, and two types of non-alloy quality steels under nine different strain asynchronous loading paths were tested by Pejkowski and Skibicki [30]. The results show that the asynchronous loading paths cause nonproportional hardening, which has a significant effect on fatigue failure life. The small cracks were observed [31], and they found that cracks initiate on the maximum shear strain planes and change direction to the maximum normal strain planes.

In addition, when dealing with asynchronous loading problems, the commonly used method is to treat the asynchronous loading path as a loading block [32] and the fatigue life is defined as the number of loading block repetitions, thus, the multiaxial cycle counting method should be used. The rainflow cycle counting method [33] is the most well-known method, and reliable results can be obtained under uniaxial loading. Lee et al. [34] verified the applicability of the rainflow cycle counting method by four steel materials using five types of triangular waveforms under asynchronous loading paths. The rainflow cycle counting method was carried out on normal and shear strain loading paths, respectively, to accumulate fatigue damage. The fatigue cycles depend on which individual strain produces more damage. However, the applicability of the rainflow cycle counting method is limited for the asynchronous loading. To overcome this problem, two cycle counting methods were proposed: the Bannantine-Socie (BS) method [35,36] and Wang-Brown (WB) method [37,38,39]. The BS method [35,36] is based on the uniaxial rainflow cycle counting method and critical plane criterion. In this method, a tensile or shear damage parameter on the critical plane is chosen, and then the number of loading reversals is accounted for. However, only the tensile or shear stress/strain is considered, and the interaction between the two parameters is ignored. The WB method [37,38,39] is based on the rainflow cycle counting method and the von Mises equivalent stress or strain. In this method, the effect of the loading history on fatigue damage accumulation may be ignored. Meggiolaro and de Castro [40,41] modified the WB method and expanded the stresses or strains into the five-dimensional Euclidean space. To overcome the drawback of the WB model, Wei and Dong [42,43,44] proposed the concept of path dependent effective stress, and the maximum range of path dependent effective stress was selected as the first reversal cycle count. In the Shang and Wang method [45], the shear stress or strain was counted by the rainflow cycle counting method on the critical plane. Anes and co-authors [46,47] proposed a virtual cycle counting method, which is a nonrainflow cycle counting method, and it is based on the stress scale factor [48]. The model was validated for 42CrMo4, 1050QT and 304L steel under 11 types of asynchronous strain loading paths. Janssens [49] proposed a universal cycle counting method independent of the damage criterion, but the method was not verified by experimental data. Multiaxial fatigue tests were performed by Arora et al. [50] to verify the applicability of the fatigue life prediction model of BS method and Smith-Watson-Topper (SWT) criterion [51] by the experimental results of SA 333 Gr. 6 steel under asynchronous loading of four different frequency ratios, however, the prediction results were non-conservative compared with the experimental fatigue life.

The limited research above indicates that the present studies were mainly focused on fatigue limits and the direction of crack initiation under asynchronous loading. The effect of frequency ratios on fatigue life and surface crack growth paths and the failure mechanism under asynchronous loading are still insufficient and further research is needed. The present study aims to investigate the effect of frequency ratios on fatigue life and surface crack growth behavior, highlighting the crack initiation and propagation path of 30CrMnSiA steel. Multiaxial fatigue experiments under asynchronous loading paths were performed, and the surface crack growth paths were observed. The mechanisms of initiation and propagation and the transformation from stage I to stage II crack were discussed with the stress analysis. The surface crack lengths versus loading blocks under asynchronous loadings were compared and analyzed. A variety of fatigue failure model including commonly used cycle counting methods, fatigue failure criteria, and Palmgren-Miner’s cumulative damage rule were used to predict fatigue life.

## 2. Materials and Methods

### 2.1. Materials

**Figure 1:**
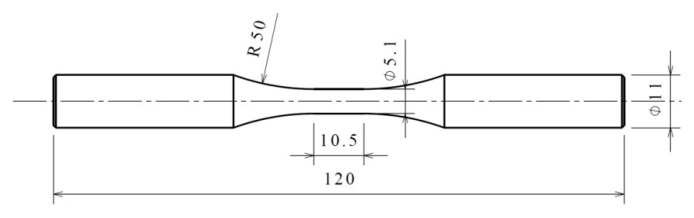
Shape and dimensions of specimens (unit: mm).

30CrMnSiA steel is the experimental material. Its chemical composition, weight percent, and mechanical properties have been given in a previous study by the authors and are also shown in Table 1 and Table 2 [6]. The geometry and dimensions of solid cylindrical bar specimens are shown in Figure 1.

### 2.2. Experimental Methods

The multiaxial fatigue tests were performed under load control mode and conducted on an MTS 858 testing system at room temperature and atmosphere. The loading frequency of a loading block used in the test was 0.5 Hz.

**Figure 2:**
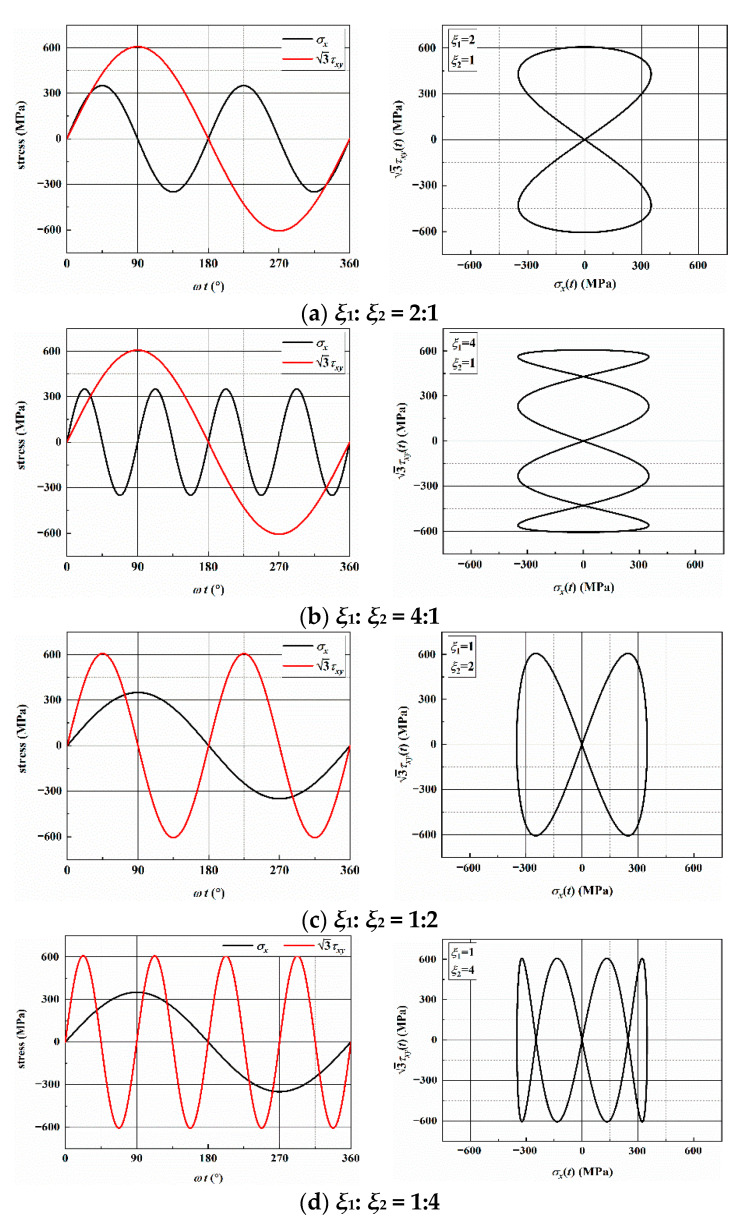
Multiaxial asynchronous loading paths with different frequency ratios.

The tension-torsion stress components of multiaxial asynchronous fatigue loading are as follows

(1) $$\sigma_{x}\left(t\right)=\sigma_{x,a}\sin\left(\xi_{1}\omega t\right),$$

(2) $$\tau_{xy}\left(t\right)=\tau_{xy,a}\sin\left(\xi_{2}\omega t\right),$$

where *σ_x_*(*t*) and *τ_xy_*(*t*) are the cyclic axial and shear stress, *σ_x_*_,*a*_ and *τ_xy_*_,*a*_ are the axial and shear stress amplitude. The loading frequency is controlled by *ξ*_1_ and *ξ*_2_. In the tests, *σ_x_*_,*a*_ = *τ_xy_*_,*a*_ = 350 MPa, that is, the stress amplitude ratio was 1.0 without a phase angle. The tests were conducted under four different frequency ratios (*ξ*_1_:*ξ*_2_) including: 2:1, 4:1, 1:2, and 1:4. The multiaxial asynchronous fatigue loading paths in a loading block with different frequency ratios are shown in Figure 2.

Each specimen was tested up to six times under the same loading conditions, and the surface crack morphologies were observed using a Zeiss metallurgical microscope with a maximum amplification capacity of 500×. The lens can be moved transversally and vertically. During the test process, the specimen was removed from the testing machine after a certain number of blocks each time, the crack morphology was observed and recorded by using the metallurgical microscope. To ensure the consistency of the test, marks were made on the clamping elements of the testing machine and the clamped end of the specimen, the specimen gage region (the central portion with equal cross section) should be avoided. In addition, cracks may initiate anywhere on the surface of the smooth tubular specimens, consequently, it is necessary to observe each position of the specimen gage region. The observation of the axial direction of the specimen was achieved by moving the lens vertically, and the observation of the circumferential direction of the specimen was achieved by rotating the specimen.

## 3. Results and Discussion

### 3.1. Multiaxial Fatigue Life

Under multiaxial asynchronous loadings, the fatigue failure life (or blocks) corresponds to the complete failure of the specimens and is defined as the number of loading blocks. The logarithmic mean fatigue life can be expressed as:

(3) $$N_{50}=10^{\left(\sum_{i=1}^{n}\log N_{f}\right)/n},$$

where *N_f_* is the fatigue failure life (or blocks).

**Figure 3:**
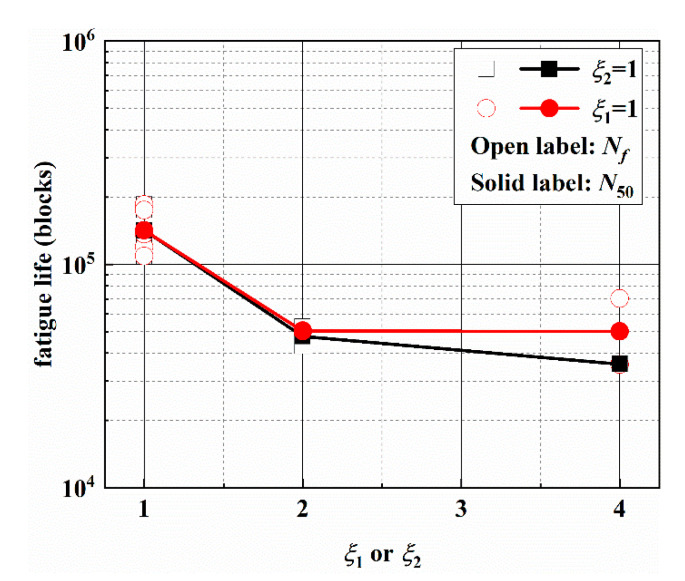
Frequency ratios effect on multiaxial fatigue life.

A total of 10 specimens were tested to ensure that there are at least 2 valid test results under each loading path. The experimental fatigue lives under different asynchronous loadings are listed in Table 3, and the test results fall in the 2 times scatter band of fatigue life. The effect of frequency ratios on the multiaxial fatigue life is given in Figure 3. For *ξ*_1_: *ξ*_2_ = 1:1, the experimental results can be found in the authors’ previous study [6]. The fatigue life decreases when *ξ*_1_ or *ξ*_2_ increase. For the condition of *ξ*_2_ = 1, fatigue life decreases significantly when *ξ*_1_ increases from 1 to 2, while it decreases by 25% when *ξ*_1_ increases from 2 to 4. For the condition of *ξ*_1_ = 1, fatigue life also decreases significantly when *ξ*_2_ increases from 1 to 2, while fatigue life has no obvious change with the increase of *ξ*_2_ from 2 to 4.

### 3.2. Stress Analysis of Individual Loading Blocks

**Figure 4:**
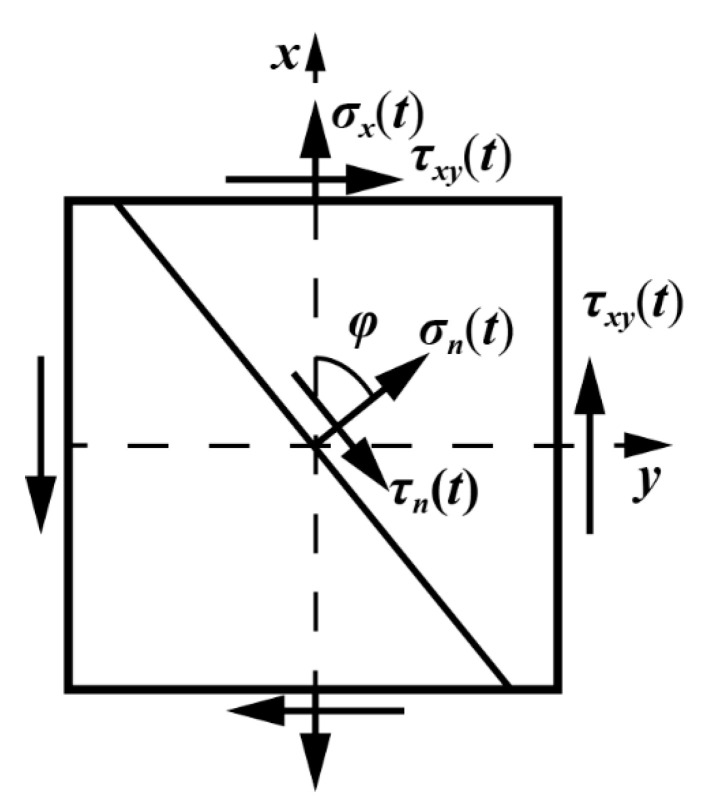
Stress state and plane direction under multiaxial fatigue loading.

Under multiaxial asynchronous loading, Figure 4 shows the stress components on an arbitrary plane, and the plane orientation is defined as *φ*. The *x* axis is consistent with the specimen axis.

Under different frequency ratios, the stress components on a plane are calculated as

(4) $$\sigma_{n}\left(t\right)=\sigma_{x,a}\cos^{2}\varphi \sin\left(\xi_{1}\omega t\right)+\tau_{xy,a}\sin2\varphi \sin\left(\xi_{2}\omega t\right),$$

(5) $$\tau_{n}\left(t\right)=-\frac{\sigma_{x,a}}{2}\sin2\varphi \sin\left(\xi_{1}\omega t\right)+\tau_{xy,a}\cos2\varphi \sin\left(\xi_{2}\omega t\right),$$

where *σ_n_*(*t*) and *τ_n_*(*t*) are the cyclic normal and shear stress on an arbitrary plane.

**Figure 5:**
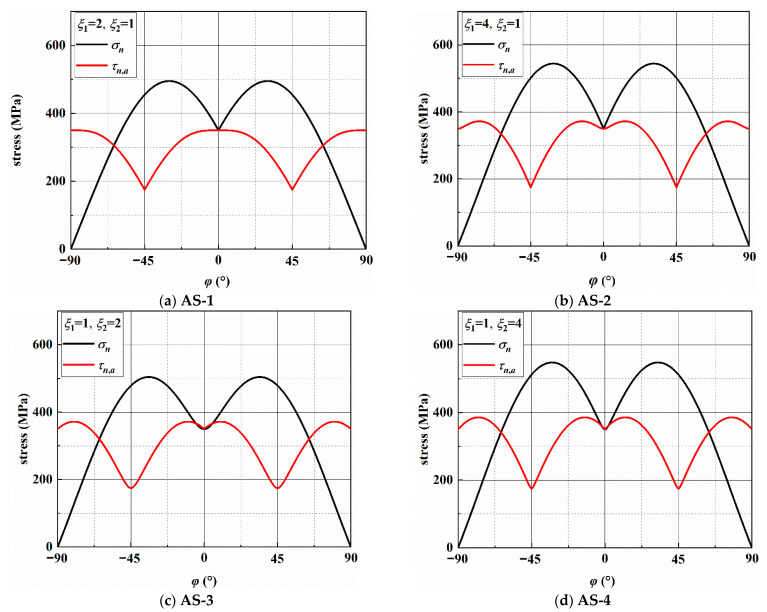
Variation in the stress components on different planes.

Under different frequency ratios, Table 4 shows the directions of the maximum shear stress amplitude (MSSA) and maximum normal (MN) planes, and the stresses on the two planes. In addition, the MN and MSSA on an arbitrary plane in a loading block are analyzed and given in Figure 5. There are four MSSA planes and two MN planes exist for each of the four frequency ratios in a loading block. In addition, it should be noted that there are two MSSA planes for AS-1. Two MN planes are axisymmetric about the axis of the specimen, and the values of *τ_n_*_,*a*_ on the two MN planes are equal.

**Figure 6:**
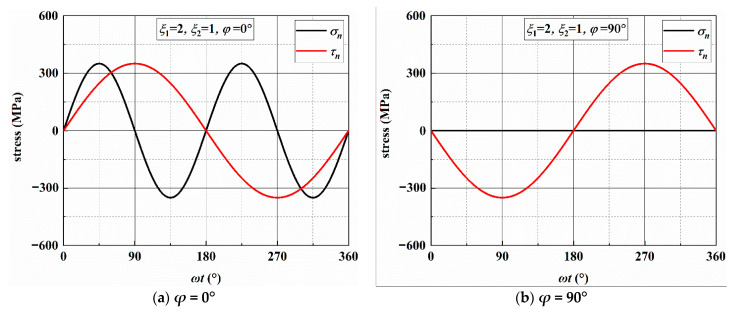
Variation in stresses on the MSSA plane under AS-1.

**Figure A1:**
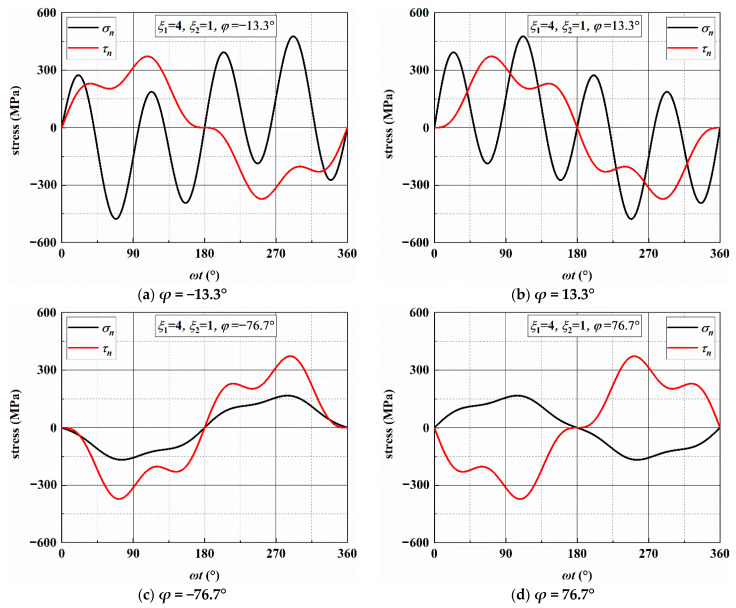
Variation of normal and shear stress on MSSA plane under AS-2.

**Figure A2:**
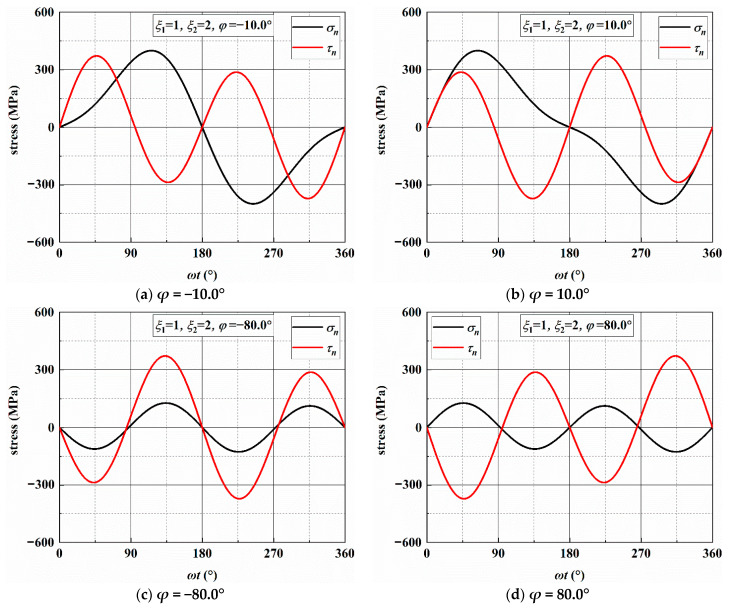
Variation of normal and shear stress on MSSA plane under AS-3.

**Figure A3:**
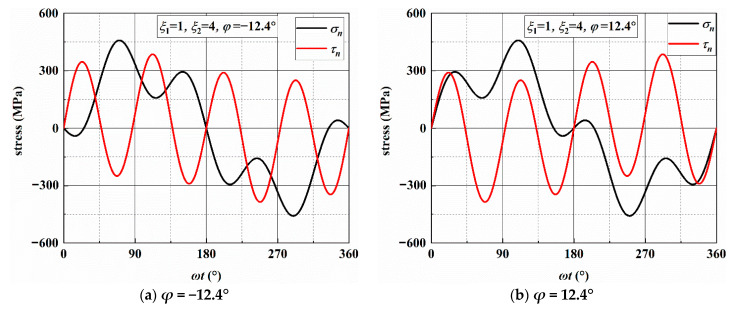

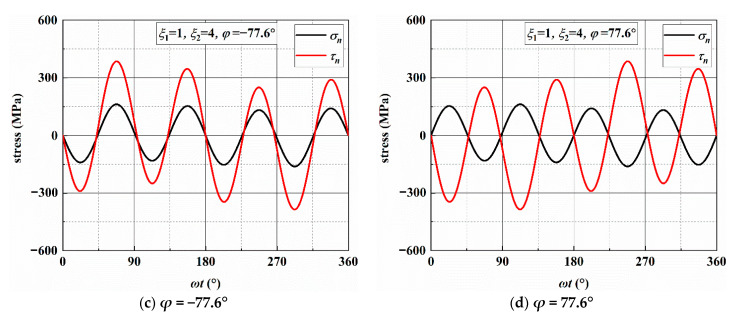
Variation of normal and shear stress on MSSA plane under AS-4.

According to previous research [6], the crack growth behavior is related to the stresses on the MSSA and MN planes. Figure 6 and Figure A1, Figure A2 and Figure A3 show the stresses on the MSSA planes under four loading paths. In Figure 6a,b, there are two MSSA planes, which are perpendicular and parallel to the direction of the specimen axis, respectively, when *ξ*_1_ = 2 and *ξ*_2_ = 1. There is only one shear stress cycle on each MSSA plane in a loading block. For normal stress, there are two cycles on the 0° plane, and the value of *σ_n_* is zero on the 90° plane. Under AS-2 with *ξ*_1_ = 4 and *ξ*_2_ = 1, the directions of the MSSA planes are ±13.3° and ±76.7°. Each MSSA plane contains three shear stress cycles in a loading block. There are four normal stress cycles on the planes of ±13.3°, and only one normal stress cycle with small stress on the planes of ±76.7°, as shown in Figure A1. When *ξ*_1_ = 1 and *ξ*_2_ = 2, there are two shear stress cycles on each plane in a loading block. However, it contains one larger normal stress cycle on the plane of ±10.0° and two smaller normal stress cycles on the plane of ±80.0°, as shown in Figure A2. For the condition of *ξ*_1_ = 1 and *ξ*_2_ = 4, each of the four MSSA planes contains four normal and shear stress cycles, respectively, in a loading block. The values of MN on the plane of ±12.4° are larger than those on the plane of ±77.6°, as shown in Figure A3.

**Figure 7:**
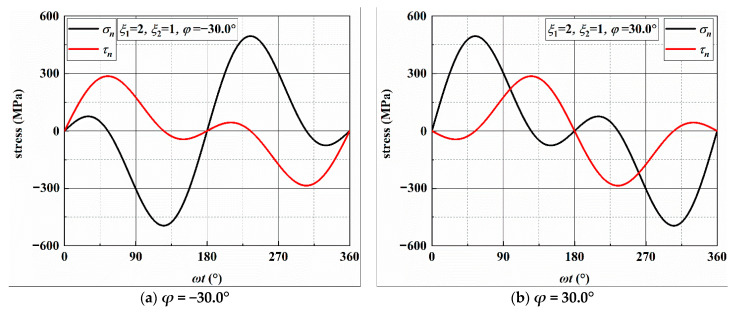
Variation in stresses on the MN plane under AS-1.

**Figure A4:**
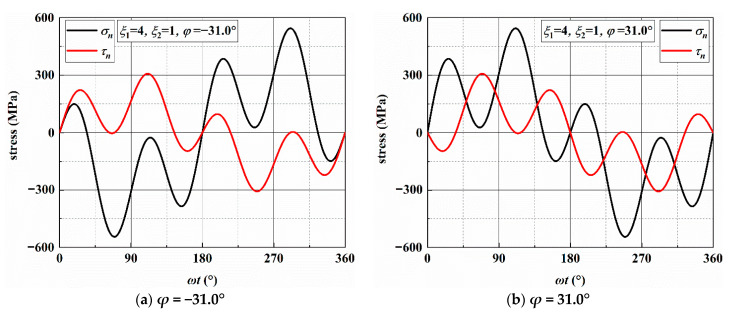
Variation of normal and shear stress on MN plane under AS-2.

**Figure A5:**
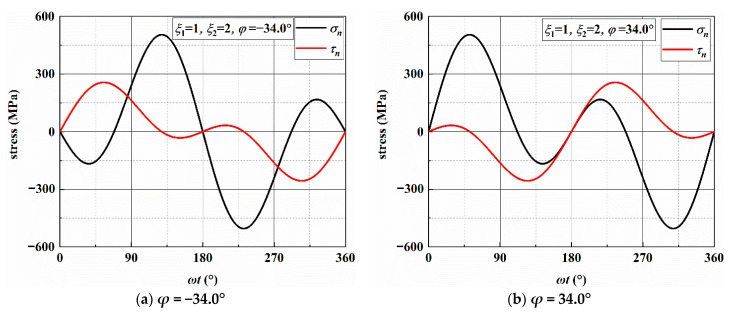
Variation of normal and shear stress on MN plane under AS-3.

**Figure A6:**
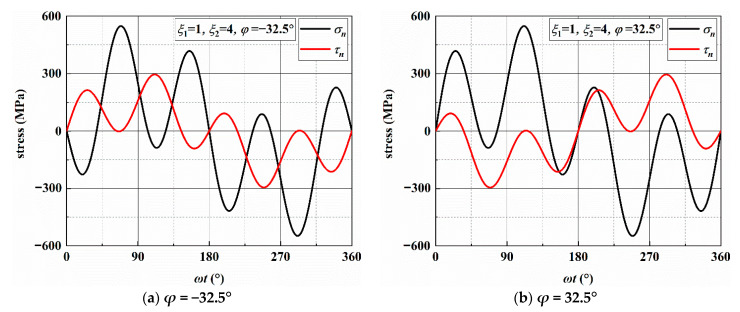
Variation of normal and shear stress on MN plane under AS-4.

Under the four loading paths, the stresses on the MN planes are calculated and given in Figure 7 and Figure A4, Figure A5 and Figure A6. There are two normal and shear stress cycles on each MN plane in a loading block for the conditions of AS-1 and AS-3 and four normal and shear stress cycles for the conditions of AS-2 and AS-4.

### 3.3. Crack Initiation and Propagation

#### 3.3.1. Crack Growth Paths of AS-1

**Figure 8:**
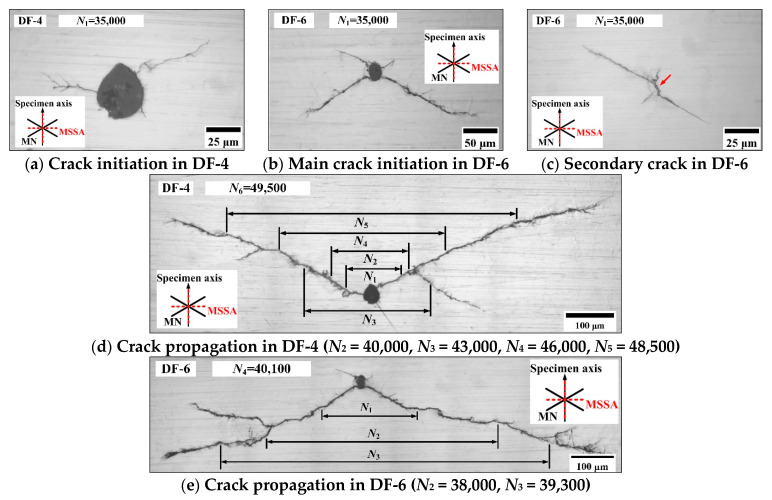
Crack morphologies in specimens DF-4 and DF-6.

For the condition of *ξ*_1_ = 2 and *ξ*_2_ = 1, the crack morphologies of specimens DF-4 and DF-6 were observed and are shown in Figure 8. The main surface cracks of the two specimens initiate on the edge of defects; both the defects are spherical, and the diameters are approximately 34 μm for DF-4 and 21 μm for DF-6, respectively. The crack growth lengths of DF-4 on the left and right sides of the defect are approximately 30 and 55 μm at 35,000 blocks (66.5% of *N_f_*). The crack propagation direction is close to the MN planes on the right side of the defect, while it is close to the MSSA plane and several small cracks branch into the direction of MN planes on the left side. For specimen DF-6, Figure 8b shows that there are four initiation cracks along the MN planes at 35,000 blocks (81.4% of *N_f_*). A secondary crack in DF-6 was also observed, as shown in Figure 8c. The stage I crack initiation on the MSSA plane and stage II cracks propagation along the MN planes were both observed. The length of stage I crack is approximately 10 μm and shorter than the diameter of defect. Consequently, the cracks initiation from the defect of DF-6 are the stage II cracks along the MN planes. The crack morphologies of DF-4 and DF-6 are similar, and the cracks propagate along the MN planes. There are two MN planes where the normal stress and shear stress amplitude are both equal, thus, the cracks branch along the MN planes of both DF-4 and DF-6 on the left and right sides of the defects as the main cracks propagate.

#### 3.3.2. Crack Growth Paths of AS-2

**Figure 9:**
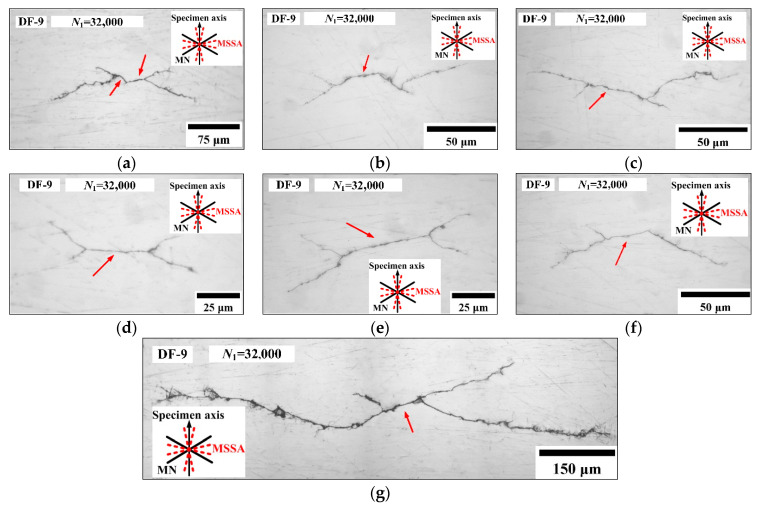
Secondary cracks (**a**–**f**) and main crack (**g**) in specimen DF-9.

For the condition of *ξ*_1_ = 4 and *ξ*_2_ = 1, at 32,000 blocks (89.6% of *N_f_*), six secondary cracks in specimen DF-9 were observed and the morphologies are shown in Figure 9a–f. For these secondary cracks, the initiation directions are all close to the MSSA planes, which are ±13.3° and bear larger normal stresses. No initiation cracks were observed on the MSSA planes of ±76.7°, this is because the normal stress on ±13.3° planes are both 476.59 MPa and larger than that on the ±76.7° plane with the value of 167.43 MPa. The lengths of stage I cracks are approximately 40–50 μm, and then the cracks propagate along the MN planes. As shown in Figure 9g, the stage Ι crack with length of 65 μm is observed for DF-9, and then the direction of crack propagation changes and branches along the two MN planes. After that, the direction of crack propagation is perpendicular to the specimen axis.

#### 3.3.3. Crack Growth Paths of AS-3

**Figure 10:**
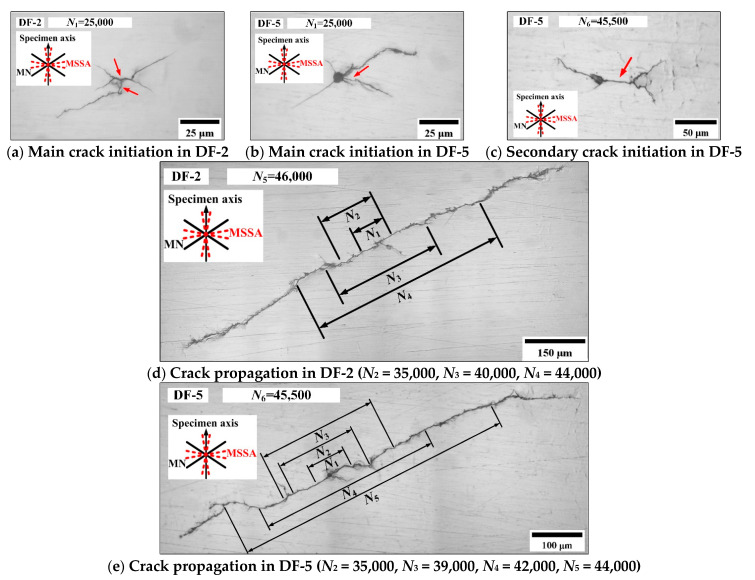
Crack morphologies in specimens DF-2 and DF-5.

For the condition of *ξ*_1_ = 1 and *ξ*_2_ = 2, Figure 10 shows the crack morphologies of DF-2 and DF-5. For DF-2 at 25,000 blocks (50.1% of *N_f_*), the surface crack initiates on the MSSA planes with larger normal stress and propagates along the MN planes; however, the length of the stage I crack is short and only approximately 10 μm. For DF-5 at 25,000 blocks (49.5% of *N_f_*), the main crack initiates on the edge of the defect and propagates along the MN planes. Otherwise, one secondary crack of DF-5 with a length of 150 μm was also observed at 45,500 blocks (90.1% of *N_f_*). The transformation from stage I to stage II crack was observed. The secondary crack initiates on the MSSA plane with a length of approximately 35 μm and propagates along the MN planes. Figure 10d,e show the main crack propagation morphologies of specimens DF-2 at 46,000 blocks (92.2% of *N_f_*) and DF-5 at 45,500 blocks (90.1% of *N_f_*), respectively, and they propagate along the MN planes. For DF-5, the defect has little effect on the fatigue life. In [52], the traditional theories based on stress concentration factors are not applicable to the small defect, and the small defect problem should be treated as the small-crack problem. Thus, the stress intensity factors rather than stress concentration factors are suggested to deal with the small defect problem.

#### 3.3.4. Crack Growth Paths of AS-4

**Figure 11:**
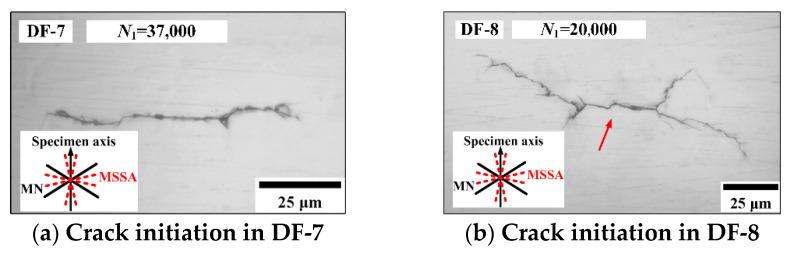

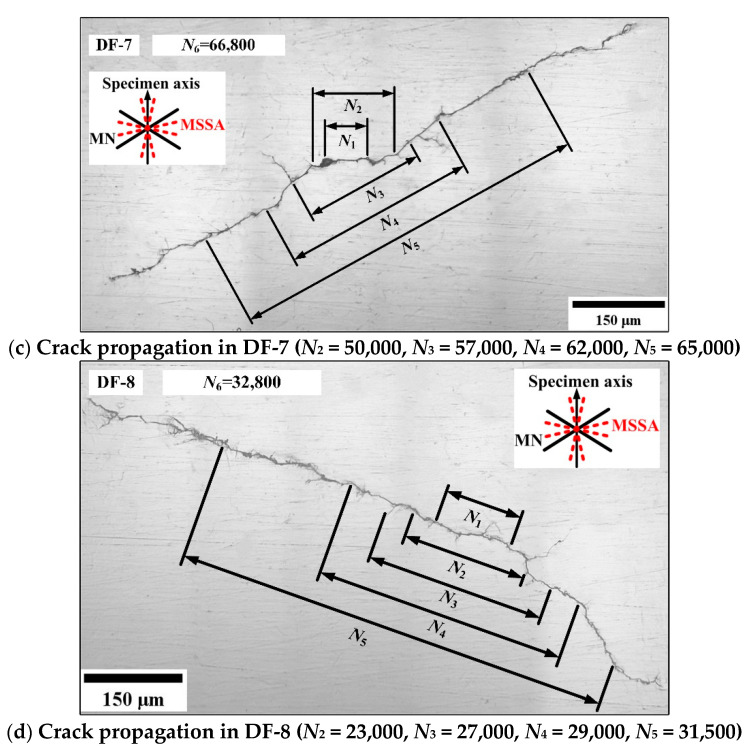
Crack morphologies in specimens DF-7 and DF-8.

For the condition of *ξ*_1_ = 1 and *ξ*_2_ = 4, the crack morphologies of DF-7 and DF-8 are shown in Figure 11. For specimen DF-7 at 37,000 blocks (52.6% of *N_f_*), only a stage I crack initiation along the MSSA plane was observed. For specimen DF-8 at 20,000 blocks (56.2% of *N_f_*), crack initiation along the MSSA plane with a length of 35 μm and propagation along the MN planes were both observed. Figure 11c,d show the main crack propagation morphologies of specimens DF-7 and DF-8, respectively. The cracks in specimens DF-7 and DF-8 propagate along the MN planes of 32.5° and −32.5° with the equal normal and shear stresses, respectively. For the specimen DF-7, the crack gradually changes direction at 5000 blocks (71.1% of *N_f_*), and the length of the stage I crack is approximately 100 μm, which is obviously longer than that in DF-8. In addition, the branch cracks propagation along MN planes are also observed in specimens DF-7 and DF-8.

**Figure 12:**
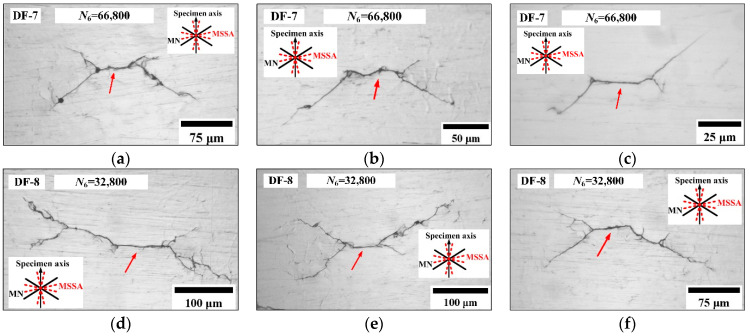
Secondary crack morphologies in specimens DF-7 (**a**–**c**) and DF-8 (**d**–**f**).

Under AS-4, Figure 12 shows that several secondary cracks were observed in DF-7 at 66,800 blocks (95.0% of *N_f_*) and in DF-8 at 32,800 blocks (92.2% of *N_f_*). The directions of secondary crack initiation and propagation are almost consistent with those of the main cracks. Otherwise, the branch cracks are also observed, and propagate along the MN planes.

As discussed above, under multiaxial asynchronous fatigue loadings with different frequency ratios, the surface stage I crack initiates on the MSSA plane with a large normal stress and the stage II cracks mainly propagate along the MN planes. For some of the loading paths, since the normal and shear stresses are both equal on the two MN planes, the cracks branching along the MN planes were observed during the main crack propagation. As the tension or torsion frequency ratio increases, more secondary cracks can be found on surface of the specimen. This may be due to the increase of shear stress amplitude and shear stress cycles in a loading block. In a previous study [6], the authors found that for pure torsional loading, high stress level will lead to more initiation of small cracks and the fatigue failure under high stress level is caused by the connection of small cracks. Under the same loading path, the morphologies of the main crack and secondary cracks are similar. For the condition where *ξ*_1_ = 2 or *ξ*_1_ = 4 and *ξ*_2_ = 1, once the crack initiates, it will propagate rapidly.

### 3.4. Crack Length versus Loading Blocks

**Figure 13:**
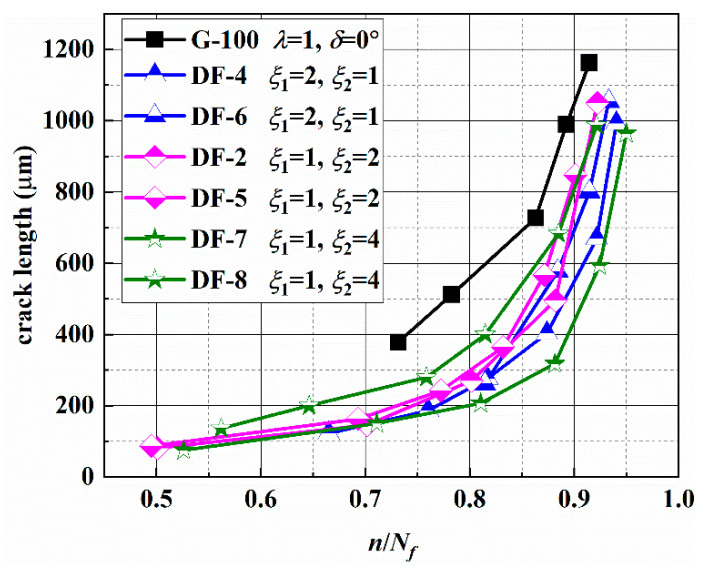
Crack length under different frequency ratios.

The main surface crack lengths versus loading blocks of different specimens were recorded and are given in Table A1 and Figure 13. The test results for G-100 under constant amplitude loading path can be found in a previous study by the authors [6]. From Figure 13, it can be seen that the crack growth length is longer for the constant amplitude loading than that for the asynchronous loading under the same fatigue life ratio. That is because that the crack length shown in the figure is a stage II-mode I crack, and the crack propagation is determined by the stress state on the MN planes. According to the stress analysis in Section 3.2, there are two different normal and shear stress cycles on each MN plane in a loading block for the conditions of ξ_1_:ξ_2_ = 2:1 and ξ_1_:ξ_2_ = 1:2 and four different normal and shear stress cycles for the conditions of ξ_1_:ξ_2_ = 4:1 and ξ_1_:ξ_2_ = 1:4. Difference between the load cycle amplitudes may lead to the crack retardation effect. However, the stage II crack propagation is not affected by the above factors for the constant amplitude loading. In addition, it can also be seen from Figure 13 that the crack growth life of stage II accounts for more than 50% of *N_f_*. When the crack length is approximately 500 μm, the crack propagation life accounts for more than 85% of *N_f_*.

### 3.5. Fatigue Life Prediction

**Figure 14:**
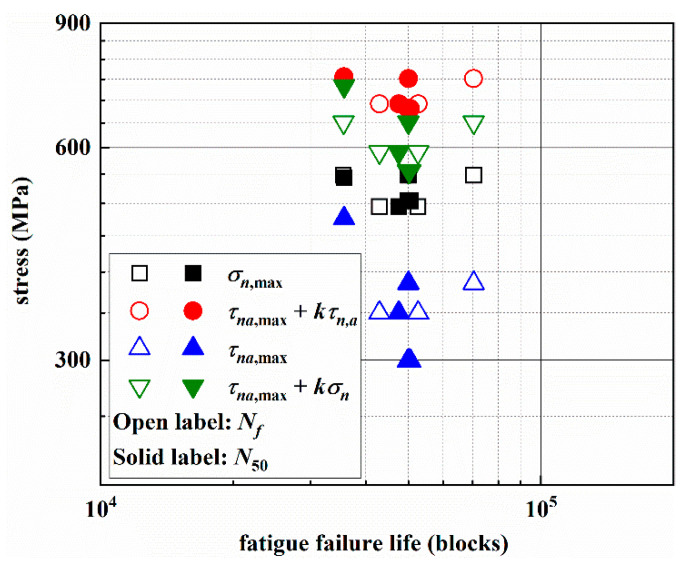
Relationship between stresses and the fatigue failure life.

From the crack growth morphologies, the stage I crack initiates along on the MSSA plane with larger normal stress, and stage II crack propagates along one of the MN planes. Therefore, the stress components on these two planes are the main factors affecting the fatigue failure life. The relationship between shear stress, normal stress, the combination of the shear stress and normal stress and the fatigue life are given in Figure 14, it shows no obvious relationship. In fact, for the multiaxial asynchronous loading paths, it should be noted that one load block may contain several different fatigue cycles. Therefore, a cycle counting method must be used to predict fatigue life under multiaxial asynchronous loads.

#### 3.5.1. Existing Multiaxial Cycle Counting Method

The counting process is more complex due to the coupling effect of tensile and shear stresses under multiaxial variable amplitude loading. Efforts have been made to apply the uniaxial cycle counting method to predict the multiaxial fatigue life. BS and WB methods are commonly used in dealing with multiaxial fatigue loadings [46,47].

To identify multiaxial fatigue load reversals, Bannantine and Socie [35,36] used the rainflow cycle counting method and critical plane criterion. Whether the tensile or shear critical plane damage parameter is chosen depends on the failure mode. When the tensile stress is selected for cycle counting, the Smith-Watson-Topper [51] damage model (SWT) is used and expressed as

(6) $$\underset{\varphi }{\max}\left\{\sigma_{n,\max}\cdot \varepsilon_{n,a}\right\}=\frac{{\sigma^{\prime }}_{f}^{2}}{E}{\left(2N_{f}\right)}^{2b}+{\sigma^{\prime }}_{f}{\varepsilon^{\prime }}_{f}{\left(2N_{f}\right)}^{b+c},$$

where *ε_n_*_,*a*_ is the principal strain amplitude, *σ’_f_* and *ε’_f_* are the axial and shear fatigue strength coefficient, *b* and *c* are the axial fatigue strength and ductility exponent.

When the shear stress is selected to count the cycle, the Fatemi-Socie [53] damage model (FS) is usually used and expressed as

(7) $$\underset{\varphi }{\max}\left\{\gamma_{na,\max}\left(1+k\frac{\sigma_{n,\max}}{\sigma_{y}}\right)\right\}=\frac{{\tau^{\prime }}_{f}}{G}{\left(2N_{f}\right)}^{b_{0}}+{\gamma^{\prime }}_{f}{\left(2N_{f}\right)}^{c_{0}},$$

where *k* is the material parameter, *γ_na_*_,max_ is the maximum shear strain amplitude, *σ_n_*_,max_ is the maximum normal stress, *τ’_f_* are the shear fatigue strength and ductility coefficient, *b*_0_ and *c*_0_ are the shear fatigue strength and ductility exponent.

**Figure A7:**
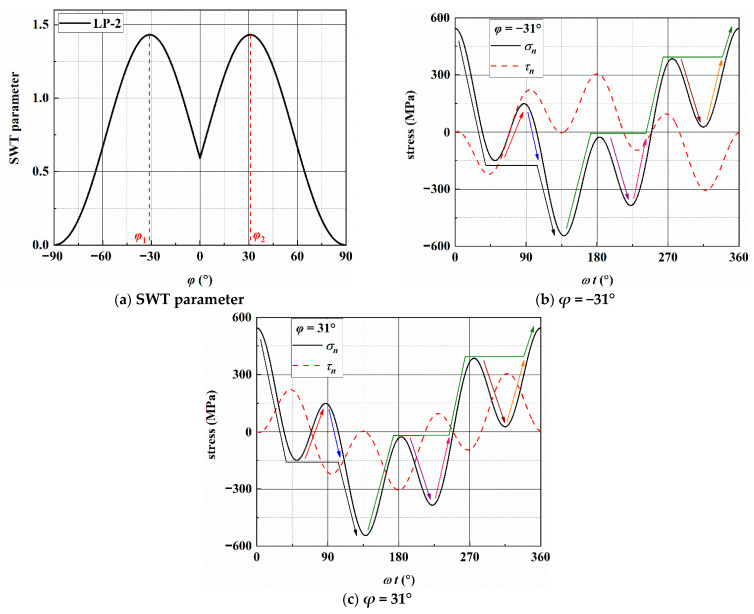
The procedure and results of BS with SWT method for reversal extraction under LP-2.

An example of the BS with the SWT method to extract the reversal counting cycles under AS-2 is shown in Figure A7.

The WB method [37,38,39] is based on rainflow cycle counting and the von Mises equivalent strain/stress to identify all reversals. Then, the damage can be calculated by the critical plane criterion or a modified strain-based multiaxial fatigue criterion due to Wang and Brown [54], as shown in Equation (8), to obtain the total fatigue life. The Wang and Brown criterion can be expressed as

(8) $$\frac{\gamma_{na,\max}+2S\varepsilon_{n,a}}{1+\nu^{\prime }+\left(1-\nu^{\prime }\right)S}=\frac{{\sigma^{\prime }}_{f}-2\sigma_{n,m}}{E}{\left(2N_{f}\right)}^{b}+{\varepsilon^{\prime }}_{f}{\left(2N_{f}\right)}^{c},$$

where *ν*’ is the effective Poisson ratio, *σ_n_*_,*m*_ is the mean normal stress, *S* is the material parameter and *S* = 1.5–2.0 for steel materials.

**Figure A8:**
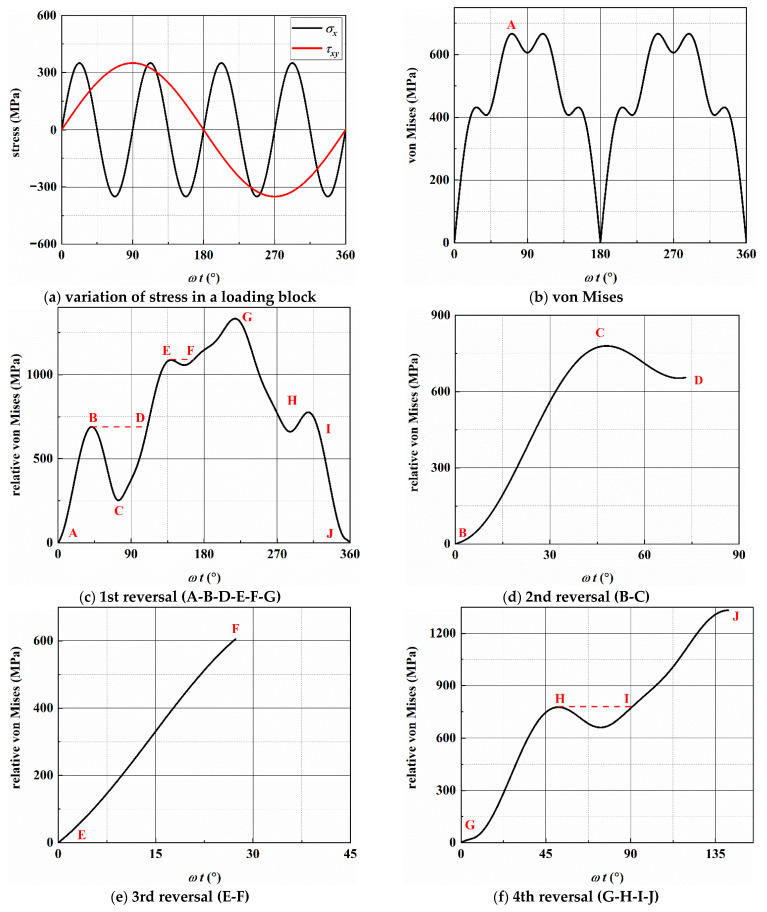

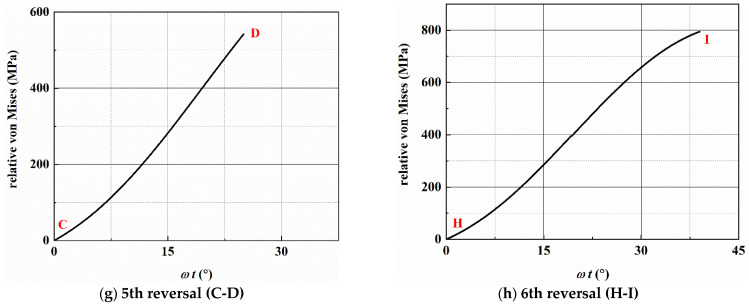
The procedure and results of WB method for reversal extraction under LP-2.

An example of using the WB method to extract the reversal counting cycles under AS-2 is given in Figure A8.

#### 3.5.2. Fatigue Life Prediction Results

The authors proposed a section critical plane method (SCPM) [55] for multiaxial high-cycle fatigue life prediction. Here it is also used for fatigue life prediction under asynchronous loadings.

To estimate the fatigue life under asynchronous loadings, the BS and WB cycle counting methods are used. Then, the fatigue life under simple cycles is predicted by the FS, SWT, WB, and SPCM criteria with the *S*-*N* curves of fully reversed uniaxial loading and fully reversed torsional loading. The *S*-*N* curves can be expressed by the following equations:

Fully reversed uniaxial tension-compression loading:

(9) $$\log N_{f}=6.9577-1.2294\log\left(\sigma_{x,a}-565.25\right).$$

Fully reversed torsional loading:

(10) $$\log N_{f}=8.8970-1.8707\log\left(\tau_{xy,a}-693.50\right).$$

**Figure 15:**
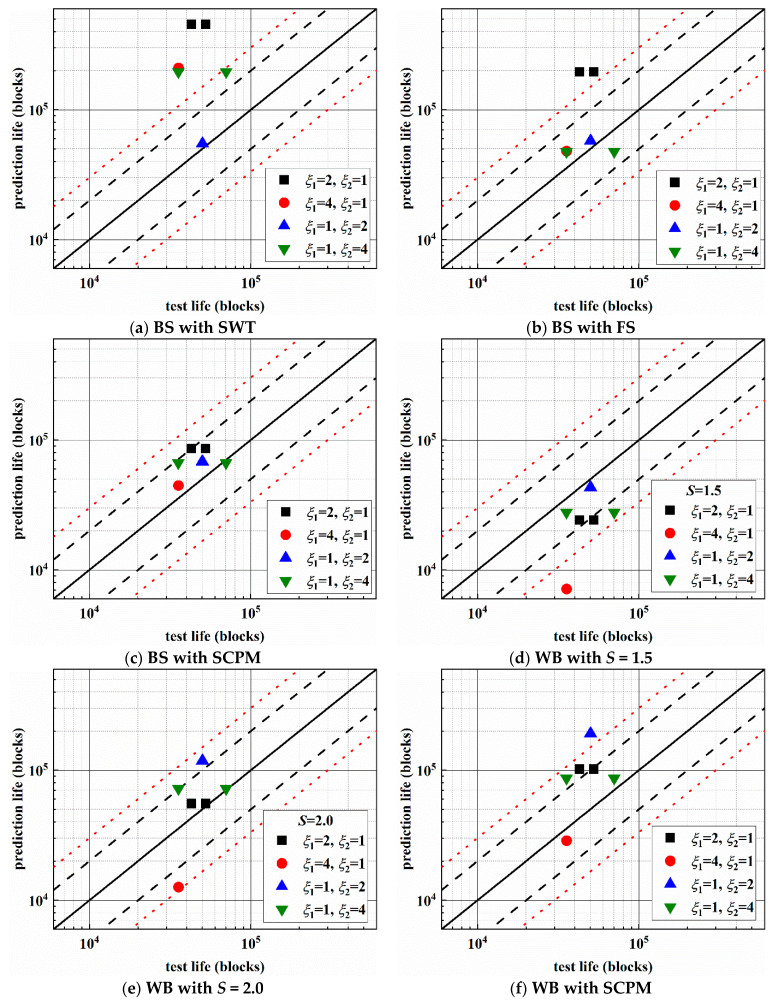
Prediction life vs. test life under different frequency ratios.

Finally, the fatigue life is obtained by the Palmgren-Miner’s linear cumulative damage rule. The comparison between the experimental and prediction results of 30CrMnSiA steel under multiaxial asynchronous loadings is given in Figure 15. The black solid line indicates that the prediction result is equal to the experimental result, the black dashed lines are used to represent the ±2 times scatter band of fatigue life, and the red dotted lines denote for the ±3 times scatter band of fatigue life. For multiaxial fatigue under stress loadings, there is no obvious engineering plastic strain. Hence only the elastic part is considered when the SWT, FS, and WB criteria are used for fatigue life prediction.

The prediction results pertaining to BS with the SWT method (mainly based on the tension stress and strain) are non-conservative and mostly exceed the 3 times range, and the discrepancies between the experimental and prediction results are large. Regarding the BS with the FS method, the prediction results lie outside of the 3 times range under AS-1, and others fall into the ±2 times range. Figure 15d,e show the results for *S* = 1.5 and *S* = 2.0, respectively. When *S* = 1.5, the prediction life is conservative and lies in the −3 times fatigue life scatter band except for AS-2. When *S* = 2.0, the prediction results all lie in the ±3 times range, and they are conservative for the condition of AS-2, while the others are non-conservative. For the two shear-based models (FS model and WB model), both the shear strain and normal strain (or stress) are taken into account, and the prediction results are much better than the SWT method. The prediction results obtained using the BS with the SCPM method all fall into the ±2 times range. Regarding the WB with the SCPM method, the prediction results lie in the ±3 times range except the condition of AS-3. The SCPM method considered the crack propagation direction under different loading paths and the prediction results are also good. For these four loading paths, when using SCPM model for life prediction, the stress components used are mainly the shear stress amplitude and normal stress on the MSSA plane.

Table 5 gives the summary of percentage in error index *Ei*. The error index *Ei* ≤ 2 and *Ei* ≤ 3 indicate the prediction results with ±2 and ±3 times scatter band of fatigue life, respectively. Compared with other models, the BS method with the SCPM criterion is more applicable for 30CrMnSiA steel under asynchronous loadings.

## 4. Conclusions

In this paper, multiaxial fatigue tests were performed on 30CrMnSiA steel under four asynchronous loadings. Subsequently, the effect of the frequency ratio on the fatigue failure life and crack behaviors were studied. As discussed above, the following conclusions can be drawn:

(a) The experimental results show that fatigue failure life under asynchronous loadings decreases when the value of ξ_1_ or ξ_2_ increase from 1 to 2, and there is no significant change when the value of ξ_1_ or ξ_2_ increase from 2 to 4.
(b) Based on the observation of the surface crack path, the crack initiates on the maximum shear stress amplitude plane with larger normal stress and propagates along the maximum normal stress planes. The proportion of the stage II crack propagation life is more than 50% of the fatigue failure life.
(c) Under asynchronous loadings, the increasing of ξ_1_ or ξ_2_ results in more shear stress cycles and larger shear stress amplitude on the MSSA planes in a loading block, and this may be the reason for the increase of secondary cracks. Furthermore, the difference between the load cycle amplitudes on the MN plane causes the crack retardation and leads to the crack growth length being longer for the constant amplitude loading than that for the asynchronous loading under the same fatigue life ratio.
(d) The applicability of Bannantine-Socie and Wang-Brown counting method is verified under multiaxial asynchronous fatigue loading with different fatigue failure criteria and Palmgren-Miner’s cumulative damage rule. The results indicate that the accuracy of the Bannantine-Socie model with the section critical plane method is higher than that of the others.

## Figures and Tables

**Table 1:** Chemical composition and weight percent of 30CrMnSiA steel (wt %). (Reproduced with permission from Liu, Crack initiation and propagation of 30CrMnSiA steel under uniaxial and multiaxial cyclic loading, published by Elsevier, 2019).

C	Mn	Si	P	Ni	Cr	W	Mo	V	Cu	Ti
0.31	0.85	0.99	0.01	0.05	0.87	0.01	0.02	0.01	0.18	0.003

**Table 2:** Mechanical properties of 30CrMnSiA steel. (Reproduced with permission from Liu, Crack initiation and propagation of 30CrMnSiA steel under uniaxial and multiaxial cyclic loading, published by Elsevier, 2019).

*E* (GPa)	*σ_y_* (MPa)	*σ_u_* (MPa)	*G* (GPa)	*τ_y_* (MPa)	*τ_u_* (MPa)
207	1196	1334	77.2	825	1040

**Table 3:** Test results under different frequency ratios.

Load Path	*σ_x_*_,*a*_ (MPa)	*τ_xy_*_,*a*_ (MPa)	*ξ*_1_	*ξ*_2_	Spec. ID	*N_f_* (blocks)	*N*_50_ (blocks)
—	350	350	1	1	G-10	185,261	141,984
					G-11	175,013	
					G-12	119,687	
					G-100	136,694	
					G-104	108,778	
AS-1	350	350	2	1	DF-4	52,632	47,560
					DF-6	42,976	
AS-2	350	350	4	1	DF-9	35,702	35,710
					DF-10	35,717	
AS-3	350	350	1	2	DF-2	49,892	50,195
					DF-5	50,500	
AS-4	350	350	1	4	DF-7	70,330	50,033
					DF-8	35,593	

**Table 4:** MSSA and MN plane orientations under different frequency ratios.

Load Path	*ξ*_1_	*ξ*_2_	MSSA	*σ_n_*/MPa	*τ_na_*_,max_ (MPa)	MN	*σ_n_*_,max_ (MPa)	*τ_n_*_,*a*_ (MPa)
AS-1	2	1	0°/90°	350/0	350.00	±30.0°	494.98	285.77
AS-2	4	1	±13.3°/±76.7°	476.59/167.43	372.16	±31.0°	544.26	307.08
AS-3	1	2	±10.0°/±80.0°	299.42/127.23	371.88	±34.0°	504.46	256.13
AS-4	1	4	±12.4°/±77.6°	385.62/161.73	385.62	±32.5°	548.07	295.18

**Table 5:** Percentage in two error indexes of each model (unit: %).

	BS-SWT	BS-FS	BS-SCPM	WB-*S* = 1.5	WB-*S* = 2.0	WB-SCPM
*Ei* ≤ 2	25.0	75.0	100.0	50.0	37.5	50.0
*Ei* ≤ 3	37.5	75.0	100.0	75.0	75.0	75.0

## Data Availability

Data are available on request to the authors.

## Nomenclature

*E*	Young’s modulus
*G*	shear modulus
*N_f_*	fatigue failure life
*N*_50_	logarithmic mean fatigue failure life
*S*	material parameter of Smith-Watson-Topper model
*k*	material parameter of Fatemi-Socie model
*b*	axial fatigue strength exponent
*b*_0_	shear fatigue strength exponent
*c*	axial fatigue ductility exponent
*c*_0_	shear fatigue ductility exponent
*ξ*_1_	axial stress frequency
*ξ*_2_	shear stress frequency
*φ*	direction of an arbitrary plane
*ν*’	effective Poisson Ratio
*σ*_*y*_	tensile yield strength
*σ*_*u*_	tensile ultimate strength
*τ*_*y*_	torsional yield strength
*τ*_*u*_	torsional ultimate strength
*σ*_*x*_(*t*)	axial stress
*σ*_*x*,*a*_	axial stress amplitude
*τ*_*xy*_(*t*)	shear stress
*τ*_*xy*,*a*_	torsion stress amplitude
*σ*_*n*_(*t*)	cyclic normal stress on the plane of *φ*
*τ*_*n*_(*t*)	cyclic shear stress on the plane of *φ*
*σ*_*n*,max_	maximum normal stress
*σ*_*n*_	normal stress on the plane of *φ*
*σ*_*n*,*a*_	normal stress amplitude on the plane of *φ*
*τ*_*n*,*a*_	shear stress amplitude on the plane of *φ*
*σ*_*n*,*m*_	mean normal stress
*τ_na_*_,max_	maximum shear stress amplitude
*γ_na_*_,max_	maximum shear strain amplitude
*ε*_*n*,*a*_	principal strain amplitude
*σ*’_*f*_	axial fatigue strength coefficient
*τ*’_*f*_	shear fatigue strength coefficient
*ε*’_*f*_	axial fatigue ductility coefficient
*γ*’_*f*_	shear fatigue ductility coefficient

## Abbreviations

AS	asynchronous loading path
MN	maximum normal
MSSA	maximum shear stress amplitude

## Appendix A

**Table A1:** Crack lengths versus loading blocks of different specimens.

Spec. ID	Contents	*N*_1_	*N*_2_	*N*_3_	*N*_4_	*N*_5_	*N*_6_
DF-4	blocks	35,000	40,000	43,000	46,000	48,500	49,500
	2*a* (μm)	122.5	186	276	403	669	993
DF-6	blocks	35,000	38,000	39,300	40,100	—	—
	2*a* (μm)	261	577	800	1054	—	—
DF-2	blocks	25,000	35,000	40,000	44,000	46,000	—
	2*a* (μm)	81	146	270	495	1050	—
DF-5	blocks	25,000	35,000	39,000	42,000	44,000	45,500
	2*a* (μm)	87	163	242	364	567	848
DF-9	blocks	32,000	—	—	—	—	—
	2*a* (μm)	920	—	—	—	—	—
DF-7	blocks	37,000	50,000	57,000	62,000	65,000	66,800
	2*a* (μm)	75	150	207	318	593	964
DF-8	blocks	20,000	23,000	27,000	29,000	31,500	32,800
	2*a* (μm)	136	200	280	400	683	987
